# Synthesis of Quaternary Heterocyclic Salts

**DOI:** 10.3390/molecules181114306

**Published:** 2013-11-19

**Authors:** Angela J. Winstead, Grace Nyambura, Rachael Matthews, Deveine Toney, Stanley Oyaghire

**Affiliations:** Department of Chemistry, Morgan State University, 1700 E Cold Spring Lane, Baltimore, MD 21251, USA

**Keywords:** quaternary ammonium salts, microwave synthesis, heterocycles

## Abstract

The microwave synthesis of twenty quaternary ammonium salts is described. The syntheses feature comparable yields to conventional synthetic methods reported in the current literature with reduced reaction times and the absence of solvent or minimal solvent.

## 1. Introduction

**Figure 1:**
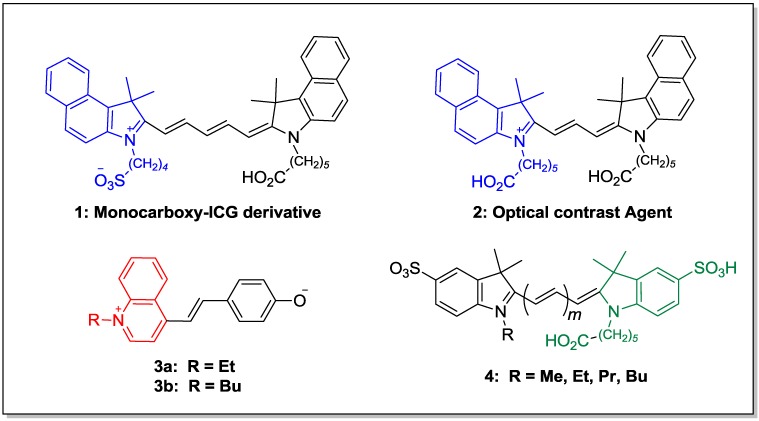
Dyes with widespread applications.

Heterocyclic chemistry has applications in diverse areas such as dyes [1], photosensitizers [2], coordination compounds [3,4], polymeric materials [5,6,7] and many other fields. Of particular interest are dyes which have widespread applications, some of which are shown in Figure 1. For example, the bone-targeting conjugated compound monocarboxy-ICG (**1**) was found to bind to growing regions of the skeleton and remain visible for two weeks [8]. The prolonged fluorescence could potentially allow for to repeated imaging with a single injection. Optical contrast agents such as cyanine **2** have been studied for ophthalmological surgery and exhibit good staining of tissues relevant for intraocular microsurgery [9]. Hydroxystyryl derivatives **3a** have been used for detecting endogenous and exogenous targets in fixed cells and tissues [10], while quinoline based merocyanines **3b** are being investigated as solvatochromic indicators [11]. Finally, sulfonated water soluble cyanine dyes **4** with reactive groups on one or both nitrogen atoms have been used extensively as fluorescence probes [12].

Cyanine dyes have equally as many uses and applications, but perhaps one of the most interesting is their advantage as fluorescent probes for biomolecular labeling, as previously stated [13,14,15]. Cyanine dyes are cationic molecules consisting of two terminal nitrogen heterocyclic subunits linked by a polymethine bridge [16]. *N*-alkyl heterocyclic quaternary ammonium salts serve as the precursor for styryl, squarine and other various cyanine dyes [17,18]. Traditionally, cyanine dyes are synthesized by a condensation reaction between two heterocyclic bases containing an activated methyl group and an unsaturated bisaldehyde in the presence of a catalyst [19].

The general procedure for symmetric cyanine dyes involves two equivalents of heterocyclic salt, one equivalent of the bisimine reagent, two equivalents of sodium acetate and solvent, which are added to a reaction vial, sealed and heated in a microwave system [20]. The ethyl cyanine dye, for example, was synthesized in 79% yield and this was confirmed by ^1^H-NMR with the disappearance of the singlet corresponding to the methyl protons of the salt at 2.87 ppm, and the appearance of the doublets of the α- and β- protons of the polymethine bridge at 6.24 ppm and 8.35 ppm, respectively. These peaks are found in the general range for all ensuing dyes.

Indolenine, benzoindolinine, quinolinium, and sulfoindolenium *N*-quaternary salts have been previously synthesized using traditional organic synthetic methods, mainly *via* refluxing the reagents with solvents such as chloroform, *o*-dichlorobenzene, acetonitrile or ethanol. Unfortunately, the reported syntheses require longer reaction times, generally 24–48 h, and results in low yields, as the process must often be repeated to achieve optimal yields.

Herein, an alternate synthetic route using microwave synthetic techniques that lower reaction times significantly is described, which produces the target benozoindolenine, quinolinium and sulfo-indolenium salts with little to no purification necessary and in comparable yield to previously reported traditional organic synthetic techniques.

## 2. Results and Discussion

### 2.1. Benzoindolenine Salts

**Scheme 1:**
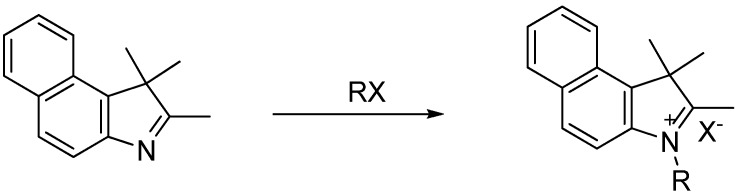
Benzoindolenine general reaction for salt derivatives.

A total of eight benzoindolenine quaternary ammonium salts were synthesized in triplicate using a Biotage Initiator 2.0 microwave oven and analyzed (Scheme 1). The optimized conditions for each heterocyclic salt were determined by conducting extensive temperature and time studies. The temperature studies for the alkyl substituted salts ranged from 120–150 °C in 10 °C increments with an initial reaction time of 5 min. The initial temperature, 120 °C, was selected based upon previous studies conducted on indolenine salts [21,22], while decomposition ensued at temperatures higher than 150 °C. Upon completion of the temperature studies, time studies were conducted which ranged from 5–45 min. Based on the temperature studies, reaction yields steadily increased from 5–30 min, while reaction times greater than 30 min resulted in decomposition. The products were purified with ether using vacuum filtration and stored in vials at room temperature under nitrogen. The structures were confirmed using proton and carbon NMR. The optimized time and temperature studies for the salts with various R-groups are summarized in Table 1.

**Table 1:** Synthesis of trimethylbenz[e]indolium deriviatives (Biotage Initiator 2.0).

Entry	R	Time (min)	Temp (°C)	Avg. Yield (%)	Lit. time (h)	Lit. yield (%)	MP (°C)
1	CH_3_	25	120	92	0.25	86 [23]	220–225
2	CH_2_CH_3_	30	135	65	24	73 [24]	225–230
3	(CH_2_)_2_CH_3_	10	140	75	N/A	N/A	142–145
4	(CH_2_)_3_CH_3_	15	140	69	20	57 [25]	120–124
5	(CH_2_)_4_CH_3_	10	145	65	1.5	24 [9]	137–139
6	(CH_2_)_2_OH	15	120	64	N/A	N/A	160–164
7	(CH_2_)_5_CO_2_H	30	145	82	48	37 [9]	174–177
8	(CH_2_)_4_SO_3_	30	145	51	2.5	70 [8]	80–85

The methyl and ethyl benzoindolenine salts exhibited similar behaviors to each other. Upon completion, both reactions resulted in solids that were isolated using vacuum filtration and cold ether washes. The methyl salt was traditionally prepared with reflux for 15 min using acetonitrile as a solvent with a 1.2 molar excess of iodomethane and was isolated with a yield of 86% using ether [23]. In this study the methyl salt was synthesized by a solvent free reaction with two equivalents of iodomethane with an increased yield of 92%. The traditional synthetic pathway of the ethyl salt consists of three lengthy steps: heating the starting material for 8 h in 1-butanol, addition of excess iodoethane and heating for 16 h, then finally recrystallization in chloroform and ether which resulted in a yield of 73% [24]. In this study, the salt was prepared in solvent free conditions with a significantly lower reaction time of 30 minutes, obtaining a 65% yield.

Propyl-, butyl-, and pentylbenzoindolenine along with the butyl indolenine salts had similar characteristics and were also synthesized in a solvent-free environment. Upon removal from the microwave, the salts appeared as liquids, however, upon addition of acetone and ether, the salts precipitated out and were easily filtered. The reaction time of the pentyl salt was 10 min and it was isolated by vacuum filtration using cold ether to yield 82% of the pure product. This is a significantly lower reaction time in comparison to traditional syntheses shown in the literature, where the time at reflux was 1.5 h, the final product was isolated by addition of ether and stirring for 16 h, and a much lower yield of 24% was achieved. Additionally, the ^1^H-NMR showed a significant amount of starting material and the product was used without further purification [9].

The carboxyl benzoindolenine salt precipitate formed immediately despite the fact that it had an increased number of carbons on the N-alkyl group. This behavior of the salt may be attributed to the effect of the polarity of the carboxylic acid has on the salt’s solubility. In the microwave system, a 1:2 ratio of the starting materials produced the pure product in a yield of 82%. This is a significant improvement in yield and reaction time compared to traditional heating, where the starting materials were refluxed in DMPU at 120 °C for 48 h with only a 37% yield [9].

Finally, the sulfobutylbenzoindolenine salt was synthesized with the starting materials in a ratio of 1:5 for 30 min in solvent free conditions. After obtaining the grey solid, the product was dried in the oven for 1 h at 100 °C. These conditions showed significant improvement over the literature, in terms of both reaction time and purification procedure. Originally, the starting materials were heated to reflux in 1,2-dichlorobenzene for 48 h and isolated by vacuum filtration with ether and later triturated in ether [8].

### 2.2. Quinolinium Salts

**Scheme 2:**
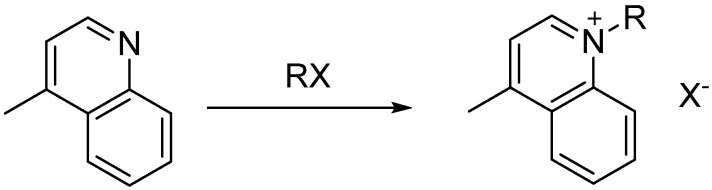
Quinolinium general reaction for salt derivatives.

A total of five quinolinium quaternary ammonium salts were successfully synthesized in the Biotage Initiator 2.0 microwave with optimal yields (Scheme 2). All reactions were performed in triplicate. The initial temperature and time settings of 150 °C and 5 min were selected based upon previous studies conducted on quinolinium salts [26]. The temperature studies for the alkyl substituted salts ranged from 130–150 °C, as any temperature above 150 °C resulted in decomposition and temperatures below 130 °C indicated significant amounts of unreacted starting material. As the carbon chain increased for each heterocyclic derivative, the reactivity consistently showed a decreasing correlation in the various reactions (Table 2).

**Table 2:** Synthesis of quinolinium deriviatives (Biotage Initiator 2.0).

Entry	R	Time (min)	Temp (°C)	Avg. Yield (%)	Lit. time (h)	Lit. Yield	MP (°C)
9	CH_3_	5	130	98	24	98 [27]	130
10	CH_2_CH_3_	5	130	70	24	59 [18]	126
11	(CH_2_)_2_CH_3_	5	130	96	N/A	80 [11]	130
12	(CH_2_)_3_CH_3_	5	130	81	5	83 [12]	125
13	(CH_2_)_5_CO_2_CH_2_CH_3_	5,2	120	56	N/A	N/A	N/A^*^

* denotes oil, boiling point not available.

The methyl, ethyl, propyl and butyl salts all exhibited similar behaviors, such as appearing as viscous brown substances initially that were precipitated by using ether and acetone. The experimental percent yield of the methyl salt was 98%, which was similar to the literature yield; however, the reaction in the literature took 24 h, a significantly higher reaction time than in the microwave, which was only 5 min [27]. Furthermore, the ethyl salt yield was 70% in 5 min in the microwave, compared to the literature where the yield was only 59% after 24 h at reflux [10].

The propyl salt yielded 96% compared to the literature yield of 80%. The butyl salt resulted in an 81% yield, however, the NMR spectrum suggested significant impurities existed after the work up, so the product was recrystallized from acetone. The pure butyl salt was a vibrant yellow color, whereas darker shades of yellow indicated impurities. In the literature, the butyl salt reaction conditions were 5 h by conventional heating in acetonitrile to produce an 83% yield [12]. The method by microwave conditions reduced the reaction time sixty-fold to only 5 min. Because of the increased carbon chain length in the 1-(6-ethoxy-6-oxohexyl)-4-methylquinolinium salt, the reaction time and temperature differed from the previous salts. The reaction was done in cycle times. Cycle times can be described as the exposure to microwave irradiation for a specific time followed by a cooling period. The concept of cycle times was introduced from a previous source [14]. The cycle times pushed the reaction to completion, after washing the product with dichloromethane and methanol, the product was concentrated and brown oil with 56% yield was obtained. 

### 2.3. Sulfoindolenium Salts

**Scheme 3:**
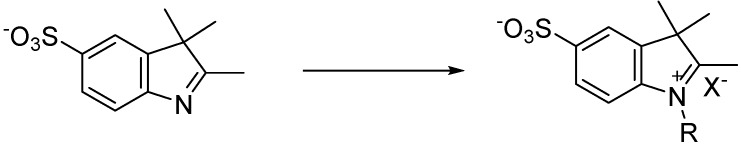
Sulfoindolenium general reaction for salt derivatives.

A total of six sulfoindolenium quaternary ammonium salts were synthesized using the CEM Discover microwave (Scheme 3). Reactions were performed in triplicate. The optimized conditions for each heterocyclic salt were determined by conducting extensive temperature and time studies. The temperature studies for the alkyl substituted salts ranged from 120–160 °C in 10 °C increments with an initial reaction time of 30 min. The reaction yields steadily increased as the temperature increased from 130–150 °C, although decomposition ensued at temperatures higher than 150 °C. Upon completion of the temperature studies, time studies were conducted, which ranged from 30 s–90 min for the alkyl substituted salts. Based on the temperature studies, the reaction yields steadily increased from 5–35 min; reaction times greater than 35 min resulted in decomposition. The hydroxyethyl salt time studies ranged from 0.5–30 min with conducted temperature studies ranging from 80–150 °C. The resulting reaction time and temperature of 5 min and 125 °C, respectively, resulted in the highest product yield. In most cases, the solid precipitate formed at room temperature with agitation. The purification methods of methyl, ethyl, propyl, and butyl were similar in terms of vacuum filtration using acetone and ether (Table 3). All salts were stored in vials at room temperature under nitrogen, and the structures were confirmed using proton and carbon NMR.

**Table 3:** Synthesis of trimethyl-5-sulfo-3*H*-indolium derivatives (Discover SP CEM).

Entry	R	Time (min)	Temp (°C)	Avg. Yield (%)	Lit. time (hr)	Lit. Yield (%)	MP (°C)
**Sulfonate**							
14	CH_3_	30	130	84	24	82 [14]	253
15	CH_2_CH_3_	30	130	67	24	95 [14]	224–229
16	(CH_2_)_2_CH_3_	30	150	63	24	98 [14]	208–211
17	(CH_2_)_3_CH_3_	35	155	58	24	96 [14]	145–149
18	(CH_2_)_2_OH	5	125	69	N/A	N/A	255–260
19	(CH_2_)_5_CO_2_H	30, 15	150	72	48	75 [14]	169–172
**Indolenine**							
20	(CH_2_)_3_CH_3_	15	140	71	24	82 [28]	105–110

Time and temperature studies were conducted with and without cycle times shown in the Table 4. The cycle is repeated a second or third time as indicated. The cycle time studies included cycle 1, which ranged from 15–90 min, and cycle 2, which ranged from 15–60 min, at a steady temperature of 150 °C. Those trials between 90–60 min resulted in a very low yield, whereas 15–30 min resulted in a higher yield during cycles 1 and 2. There was one study with three cycle times at a constant temperature which also resulted in low yield.

**Table 4:** Results from cycle *vs*. non cycles of 1-(5-carboxypentyl)-2,3,3-trimethyl-5-sulfo-3*H*-indolium salt (Entry 19).

Non cycle Time (min)	Temp. (°C)	Yields (%)
**30**	150	37
**60**	150	51
**90**	150	8
**75**	150	-
**Cycle Time (min)**	**Temp. (°C)**	**Yields (%)**
**60, 30**	150	24
**60, 60**	150	6
**30, 15**	150, 130	38
**60, 30, 15**	150	5
**90, 15**	150	43
**30, 15**	150	80

It has been stated that quaternary ammonium salts described are hygroscopic. In a previous report [29], the structure of the indolenium carboxyl salt was confirmed using X-ray crystallography. The structure showed that both ions in the salt form intermolecular hydrogen bonds with the water molecule. The O-H…Br interactions form a distorted tetrahedral array about the central Br atom.

## 3. Experimental

### 3.1. General

All microwave reactions were conducted using either the single mode Biotage Initiator 2.0 (Biotage, Uppsala, Sweden); benzoindolinine and quinolinium salts) or Discover SP CEM microwave oven (CEM Corporation, Matthews, NC, USA); sulfoindolenium salts. ^1^H and ^13^C-NMR spectra were obtained in DMSO-d_6_ (obtained from Cambridge Isotope Laboratories (Cambridge Isotope Laboratories, Tewksbury, MA, USA) using a Bruker Advance 400 MHz NMR and were recorded at 400 MHz and 100 MHz, respectively. Melting points are uncorrected. All reagents and chemicals were obtained from Aldrich Chemical Company (St. Louis, MO, USA) and Alfa Aesar (Ward Hill, MA, USA) and were used without further purification.

### 3.2. Synthesis of Trimethylbenz[e]indolium Salts

*1,2,3,3-Tetramethylbenz[e]indolium iodide* (**1**): 2,3,3-Trimethybenzolindolenine (0.30 g, 1.4 mmol) and iodomethane (0.18 mL, 3.0 mmol) were added to a microwave reaction vial. The microwave oven was set to 120 °C for 25 min with a ramp time of 2 min. After cooling, the green solid was filtered and washed with cold ether and dried *in vacuo*, yielding a grey solid in 80% yield. MP 220–225 °C; ^1^H-NMR δ (ppm): 8.38 (d, *J* = 8.5 Hz, 1H), 8.31 (d, *J* = 8.9 Hz, 1H), 8.23 (d, *J* = 7.8 Hz, 1H), 8.12 (d, *J* = 8.9 Hz, 1H), 7.78 (m, 2H), 4.11 (s, 3H), 2.89 (s, 3H), 1.76 (s, 6H); ^13^C-NMR δ (ppm): 195.8 (C), 139.4 (CH), 136.4 (CH), 132.9 (CH), 130.4 (CH), 129.7 (C), 129.3 (C), 127.0 (C), 123.3 (CH), 113.1 (CH), 55.2 C), 35.1(CH_3_), 21.2 (CH_3_), 14.0 (CH_3_).

*1-Ethyl-2,3,3-trimethylbenz[e]indolium iodide* (**2**): 2,3,3-Trimethybenzolindolenine (0.30 g, 1.4 mmol) and iodoethane (0.52 mL, 6.5 mmol) were added to a microwave reaction vial. The microwave oven was set to 135 °C for 30 min with a ramp time of 2 min. After cooling, the white-blue solid was filtered and washed with cold ether and dried *in vacuo*, yielding 80% of pure product. MP 225–230 °C; ^1^H-NMR δ (ppm): 8.39 (d, *J* = 8.3 Hz, 1H), 8.32 (d, *J* = 8.9 Hz, 1H), 8.24 (d, *J* = 8.0 Hz, 1H), 8.18 (d, *J* = 8.9 Hz, 1H), 7.78 (m, 2H), 4.64 (q, *J* = 7.3 Hz, 2H), 2.96 (s, 3H), 1.77 (s, 6H), 1.52 (t, *J* = 7.3 Hz, 3H); ^13^C-NMR δ (ppm): 195.9 (C), 138.1 (CH), 136.9 (CH), 132.9 (CH), 130.6 (CH), 129.6 (C), 128.3 (C), 127.23 (C), 127.20 (C), 123.3 (C), 113.1 (CH), 55.4 (C), 43.3 (CH_2_), 21.4 (CH_3_), 13.6 (CH_3_), 12.8 (CH_3_).

*1-Propyl-2,3,3-trimethylbenz[e]indolium iodide* (**3**): 2,3,3-Trimethybenzolindolenine (0.30 g, 1.4 mmol) and iodopropane (0.54 mL, 5.5 mmol) were added to a microwave reaction vial. The microwave oven was set to 140 °C for 10 min with a ramp time of 2 min. After cooling, the product was stirred in acetone and ether. The yellow solid was filtered and washed with cold ether and dried *in vacuo*, yielding 83% pure product. MP 142-145 °C; ^1^H-NMR δ (ppm): 8.40 (d, *J* = 8.4 Hz, 1H), 8.32 (d, *J* = 8.9 Hz, 1H), 8.24 (d, *J* = 8.0 Hz, 1H), 8.21 (d, *J* = 8.9 Hz, 1H), 7.79 (m, 2H), 4.60 (t, *J* = 7.4 Hz, 2H), 2.98 (s, 3H), 1.95 (m, 2H), 1.79 (s, 6H), 1.04 (t, *J* = 7.4 Hz, 3H); ^13^C-NMR δ (ppm): 196.4 (C), 138.5 (CH), 136.9 (CH), 132.9 (CH), 130.6 (CH), 129.6 (C), 128.3 (C), 127.2 (C), 127.1 (C), 123.4 (C), 113.3 (CH), 55.4 (C), 49.0 (CH_2_), 21.6 (CH_2_), 21.0 (CH_3_), 13.9 (CH_3_), 10.7 (CH_3_).

*1-Butyl-2,3,3-trimethylbenz[e]indolium iodide* (**4**): 2,3,3-Trimethybenzolindolenine (0.30 g, 1.4 mmol) and iodobutane (1.0 mL, 8.7 mmol) were added to a microwave reaction vial. The microwave oven was set to 140 °C for 15 min with a ramp time of 2 min. After cooling, the product was stirred in acetone and ether. The yellow solid was filtered and washed with cold ether and dried *in vacuo*, yielding 82% pure product. MP 120–124 °C; ^1^H-NMR δ (ppm): 8.40 (d, *J* = 8.3 Hz, 1H), 8.31 (d, *J* = 8.9 Hz, 1H), 8.24 (d, *J* = 7.9 Hz, 1H), 8.18 (d, *J* = 8.9 Hz, 1H), 7.77 (m, 2H), 4.61 (t, *J* = 7.7 Hz, 2H), 2.98 (s, 3H), 1.89 (m, 2H), 1.78 (s, 6H), 1.49 (m, 2H), 0.97 (t, *J* = 7.4 Hz, 3H); ^13^C-NMR δ (ppm): 196.2 (C), 138.4 (CH), 136.9 (CH), 133.0 (CH), 130.6 (CH), 129.6 (C), 128.3 (C), 127.2 (C), 123.4 (C), 113.3 (CH), 55.4 (C), 47.7 (CH_2_), 29.4 (CH_2_), 21.6 (CH_2_), 19.3 (CH_3_), 13.9 (CH_3_), 13.6 (CH_3_).

*1-Pentyl-2,3,3-trimethylbenz[e]indolium iodide* (**5**): 2,3,3-Trimethybenzolindolenine (0.30 g, 1.4 mmol) and iodopentane (1.2 mL, 9.1 mmol) were added to a microwave reaction vial. The microwave oven was set to 145 °C for 10 min with a ramp time of 2 min. After cooling, the product was stirred in acetone and ether. The orange solid was filtered and washed with cold ether and dried *in vacuo*, yielding 82% pure product. MP 137–139 °C; ^1^H-NMR δ (ppm): 8.40 (d, *J* = 8.3 Hz, 1H), 8.31 (d, *J* = 8.9 Hz, 1H), 8.24 (d, *J* = 7.9 Hz, 1H), 8.18 (d, *J* = 8.9 Hz, 1H), 7.77 (m, 2H), 4.61 (t, *J* = 7.7 Hz, 2H), 2.98 (s, 3H), 1.89 (m, 2H), 1.78 (s, 6H), 1.49 (m, 2H), 0.97 (t, *J* = 7.4 Hz, 3H); ^13^C-NMR δ (ppm): 196.2 (C), 138.4 (CH), 136.9 (CH), 132.9 (CH), 130.6 (CH), 129.6 (C), 128.3 (C), 127.2 (C), 127.1 (C), 127.0 (C), 123.4 (C), 113.3 (CH), 55.4 (C), 47.8 (CH_2_), 27.9 (CH_2_), 27.1 (CH_2_), 21.8 (CH_2_), 21.6 (CH_3_), 13.8 (CH_3_), 13.7 (CH_3_).

*1-(2-Hydroxyethyl)-2,3,3-trimethylbenz[e]indolium bromide* (**6**): 2,3,3-Trimethybenzolindolenine (0.30 g, 1.4 mmol) and 2-bromoethanol (0.2 mL, 2.8 mmol) were added to a microwave reaction vial. The microwave oven was set to 120 °C for 15 min with a ramp time of 2 min. After cooling, the product was stirred in acetonitrile, acetone and ether. The grey solid was filtered and washed with cold ether and dried *in vacuo*, yielding 74% pure product. MP 160–164 °C; ^1^H-NMR δ (ppm): 8.40 (d, *J* = 8.4 Hz, 1H), 8.30 (d, *J* = 8.9 Hz, 1H), 8.23 (d, *J* = 7.7 Hz, 1H), 8.19 (d, *J* = 8.9 Hz, 1H), 7.79 (m, 2H), 4.76 (t, *J* = 4.9 Hz, 2H), 3.95 (t, *J* = 4.8 Hz, 2H), 2.96 (s, 3H), 1.79 (s, 6H); ^13^C-NMR δ (ppm): 197.3 (C), 138.6 (CH), 136.7 (CH), 132.9 (CH), 130.4 (CH), 129.6 (C), 128.3 (C), 127.1 (C), 123.3 (C), 113.5 (CH), 57.9 (C), 55.5 (CH_2_), 50.4 (CH_2_), 21.5 (CH_3_), 14.3 (CH_3_).

*1-(5-Carboxypentyl)-2,3,3-trimethylbenz[e]indolium iodide* (**7**): 2,3,3-Trimethybenzolindolenine (0.30 g, 1.4 mmol), 6-bromohexanoic acid (0.60 g, 3.0 mmol) and 1,2-dichlorobenzene (1.5 mL 13.2 mmol) were added to a microwave reaction vial. The microwave oven was set to 145 °C for 30 min with a ramp time of 2 min. After cooling, the product was stirred in ether. The yellow solid was filtered and washed with cold ether and dried *in vacuo*, yielding 85% pure product. MP 174–177 °C; ^1^H-NMR δ (ppm): 8.40 (d, *J* = 8.2 Hz, 1H), 8.31 (d, *J* = 8.9 Hz, 1H), 8.24 (d, *J* = 8.1 Hz, 1H), 8.20 (d, *J* = 9.0 Hz, 1H), 7.77 (m, 2H), 4.62 (t, 7.5 Hz, 2H), 2.98 (s, 3H), 2.25 (t, *J* = 7.1 Hz, 2H), 1.92 (m, 2H), 1.78 (s, 6H), 1.60 (m, 2H), 1.49 (m, 2H); ^13^C-NMR δ (ppm): 196.3 (C), 174.3 (C), 138.4 (CH), 136.9 (CH), 132.9 (CH), 130.6 (CH), 129.6 (C), 128.3 (C), 127.2 (C), 127.1 (C), 123.4 (C), 113.3 (CH), 55.4 (C), 47.6 (CH_2_), 33.3 (CH_2_), 27.1 (CH_2_), 25.3 (CH_2_), 24.0 (CH_2_), 21.7 (CH_3_), 14.4 (CH_3_) 13.8 (CH_3_).

*3-(4-Sulfobutyl)-1,1,2-trimethylbenz[e]indolium iodide* (**8**): 2,3,3-Trimethybenzolindolenine (0.30 g, 1.4 mmol) and 1,4-butane sultone (0.70 mL, 6.8 mmol) were added to a microwave reaction vial. The microwave oven was set to 145 °C for 30 min with a ramp time of 2 min. After cooling, the product was stirred in acetone and ether. The purple solid was filtered and washed with cold ether and dried in an oven at 100 °C, yielding 83% pure product. MP 80–85 °C; ^1^H-NMR δ (ppm): 8.40 (d, *J* = 8.3 Hz, 1H), 8.31 (d, *J* = 8.9 Hz, 1H), 8.24 (d, *J* = 7.9 Hz, 1H), 8.18 (d, *J* = 8.9 Hz, 1H), 7.75 (m, 2H), 4.63 (t, *J* = 8.02 Hz, 2H), 2.96 (s, 3H), 2.57 (t, *J* = 7.19 Hz, 2H), 2.51 (m 2H), 2.04 (m, 2H), 1.75 (s, 6H); ^13^C-NMR δ (ppm): 196.3 (C), 138.5 (CH), 136.8 (CH), 132.9 (CH), 131 (CH), 130.6 (C), 129.6 (C), 127.1 (C), 123.3 (C), 113.4 (CH), 55.4 (C), 50.1 (CH_2_), 47.4 (CH_2_), 26.1 (CH_2_), 22.0 (CH_2_), 21.5 (CH_3_), 13.6 (CH_3_).

### 3.3. Synthesis of Quinolinium Salts

*1,4-Dimethylquinolinium iodide* (**9**): Lepidine (0.20 mL 1.4 mmol) and iodomethane (0.22 mL, 0.28 mmol) were added to a microwave reaction vial. The microwave oven was set to 130 °C for 5 min with a ramp time of 2 min. After cooling, the product was washed with cold ether and acetone and dried *in vacuo*, yielding a yellow-green solid in 98% yield. MP 130 °C; ^1^H-NMR δ (ppm): 9.36 (q, *J* = 7.33 Hz, 2H), 8.52 (t, *J* = 8.55 Hz, 1H), 8.28 (q, *J* = 8.48 Hz, 2H), 8.07 (d, *J* = 6.05 Hz, 1H), 4.58 (s, 3H), 3.41 (s, 3H); ^13^C-NMR δ (ppm): 158.0 (C), 148.8 (C), 137.5 (CH), 134.8 (CH), 129.5 (CH), 128.3 (CH), 126.6 (CH), 122.3 (CH), 119.4 (CH), 44.9 (CH_2_), 19.5 (CH_3_).

*1-Ethyl-4-methylquinolinium iodide* (**10**): Lepidine (0.20 mL, 1.4 mmol) and iodoethane (0.39 mL, 0.28 mmol) were added to a microwave reaction vial. The microwave oven was set to 130 °C for 5 min with a ramp time of 2 min. After cooling, the product was filtered and washed with cold ether and acetone and dried *in vacuo*, yielding a yellow solid in 70% yield. MP 126 °C; ^1^H-NMR δ (ppm): 9.43 (d, *J* = 7.33 Hz, 1H), 8.58 (dd, *J* = 8.55 Hz, 2H), 8.27 (t, *J* = 8.48 Hz, 1H), 8.07 (d, *J* = 6.05 Hz, 2H), 5.01 (q, *J* = 7.22 Hz, 2H), 3.01 (s, 3H), 1.59 (t, *J* = 7.22 Hz, 3H); ^13^C-NMR δ (ppm): 158.2 (C), 148.0 (C), 136.4 (CH), 134.9 (CH), 129.1 (CH), 128.8 (CH), 127.0 (CH), 122.6 (CH), 119.1 (CH), 52.4 (CH_2_), 19.5 (CH_3_), 15.1 (CH_3_).

*1-Propyl-4-methylquinolinium iodide* (**11**): Lepidine (0.20 mL, 1.4 mmol) and iodopropane (0.27 mL, 0.28 mmol) were added to a microwave reaction vial. The microwave oven was set to 130 °C for 5 min with a ramp time of 2 min. After cooling, the product was filtered and washed with cold ether and acetone and dried *in vacuo*, yielding 96% pure product. MP 130 °C; ^1^H-NMR δ (ppm): 9.6 (d, *J* = 6.05 Hz, 1H), 8.59 (q, *J* = 8.48 Hz, 2H), 8.31 (t, *J* = 8.55 Hz, 1H), 8.07 (q, *J* = 7.33 Hz, 2H), 4.98 (t, *J* = 7.39 Hz, 2H), 3.02 (s, 3H), 1.98 (m, 2H), 0.96 (t, *J* = 7.36 Hz 3H); ^13^C-NMR δ (ppm): 158.5 (C), 148.3 (C), 136.7 (C), 135.0 (CH), 129.5 (CH), 128.9 (CH), 127.1 (CH), 122.5 (CH), 119.3 (CH).

*1-Butyl-4-methylquinolinium iodide* (**12**): Lepidine (0.20 mL, 1.4 mmol) and iodobutane (0.32 mL, 0.28 mmol) were added to a microwave reaction vial. The microwave oven was set to 130 °C for 5 min with a ramp time of 2 min. After cooling, the product was filtered and washed with cold ether and acetone and dried *in vacuo*, yielding the product as yellow crystals in 81% yield. MP 125 °C; ^1^H-NMR δ (ppm): 9.6 (d, *J* = 6.05 Hz, 1H), 8.59 (q, *J* = 8.48 Hz, 2H), 8.3 (t, *J* = 8.55 Hz, 1H), 8.08 (q, *J* = 7.33 Hz, 2H), 5.0 (t, *J* = 7.49 Hz, 2H), 3.0 (s, 3H), 1.93 (m, 2H), 1.40 (m, 2H), 0.94 (t, *J* = 7.32 Hz, 3H); ^13^C-NMR δ (ppm): 158.5 (C), 148.3 (C), 136.6 (C), 135.0 (CH), 129.5 (CH), 128.9 (CH) 127.1 (CH), 122.6 (CH), 119.3 (CH), 56.7 (CH_2_), 19.7 (CH_2_), 19.1 (CH_3_), 13.4 (CH_3_).

*1-(6-ethoxy-6-oxohexyl)-4-methylquinolinium iodide* (**13**): Lepidine (0.23 mL, 1.754 mmol) and ethyl 6-iodohexanoate (0.35 mL, 1.929 mmol) were combined in a 5 mL Biotage microwave vial equipped with a stir bar. The vial was placed in the Biotage microwave with a ramp time of 4 min and held at 120 °C for 5 min. The vial with reaction mixture was allowed to sit for 5 min. and irradiated again with a ramp time of 4 min and held at 120 °C for 2 min. The resulting dark brown solid was dissolved in a mixture of ethyl acetate (5 mL) and methanol (0.5 mL). The dark brown mixture was purified by eluting on a coarse frit packed with silica gel. Ethyl acetate (400 mL) was used to wash the product and a mixture of dichloromethane and methanol (90:10) was used for subsequent washes. The dichloromethane and methanol washes were concentrated and dried to obtain brown oil in 56% yield. ^1^H-NMR δ (ppm): 9.45 (d, *J* = 6.05 Hz, 2H), 8.56 (m, 2H), 8.27 (m, 1H), 8.08 (m, 2H), 5.02 (t, *J =* 7.47 Hz, 2H), 4.02 (q, *J =* 7.11, Hz, 2H), 3.00 (s, 3H), 1.95 (m, 2H), 1.60 (m, 2H), 1.55 (m, 2H), 1.38 (m, 2H), 1.15 (t, *J =* 7.47 Hz, 3H). ^13^C-NMR δ (ppm): 172.7 (C), 158.5 (C), 148.4 (CH), 136.6 (CH), 135.0 (CH), 129.5 (CH), 128.9 (CH), 127.1 (CH), 122.6 (CH), 119.3 (CH), 59.7 (CH_2_), 56.6 (CH_2_), 33.1 (CH_2_), 29.0 (CH_2_), 25.1 (CH_2_), 23.8 (CH_2_), 19.7 (CH_3_), 14.1 (CH_3_).

### 3.4. Synthesis of Sulfoindolenium Salts

*1,2,3,3-Tetramethyl-5-sulfo-3H-indolium* (**14**): Iodomethane (0.11 mL, 2.2 mmol) and potassium 2,3,3-trimethyl-3*H*-indole-5-sulfonate (0.10 g, 0.36 mmol) in 1 mL of acetonitrile were added to a microwave reaction vial. The CEM microwave oven was set to 130 °C for 30 min with a ramp time of 1 min. After cooling, the product was boiled in acetone for 5 min and was recrystallized upon cooling. The solid was filtered and washed with acetone and cold ether and dried *in vacuo,* yielding a red solid in 84% yield. MP 253 °C; ^1^H-NMR δ (ppm): 7.96 (s, 1H) 7.84 (d, *J =* 8.34 Hz, 2H), 7.76 (d, *J =* 1.5 Hz, 2H), 3.95 (s, 3H), 2.77 (s, 3H), 2.09 (s, 6H); ^13^C-NMR δ (ppm): 195.5 (C), 148.0 (CH), 140.7 (CH), 139.9 (CH), 124.9 (C), 119.3 (C), 113.2 (C), 52.7 (C), 33.5 (CH_3_), 20.3 (CH_3_), 13.0 (CH_3_).

*1-Ethyl-2,3,3-Trimethyl-5-sulfo-3H-indolium* (**15**): Iodoethane (0.11 mL, 1.4 mmol) and potassium 2,3,3-trimethyl-3*H*-indole-5-sulfonate (0.10 g, 0.36 mmol) in 1 mL of acetonitrile were added to a microwave reaction vial. The CEM microwave oven was set to 130 °C for 30 min with a ramp time of 1 min. After cooling, the red solid was filtered and washed with acetone and cold ether and dried *in vacuo*, yielding a red solid in 67% pure product. MP 224–229 °C; ^1^H-NMR δ (ppm): 8.02 (s, 1H), 7.92 (d, *J =* 8.35 Hz, 1H), 7.82 (d, *J =* 1.5 Hz, 1H), 4.49 (q, *J =* 7.34 Hz, 2H), 2.84 (s, 3H), 2.09 (s, 6H), 1.44 (t, *J =* 7.33 Hz, 3H); ^13^C-NMR δ (ppm): 195.6 (C), 148.1 (CH), 140.3 (CH), 139.3 (CH), 125.1 (C), 119.5 (C), 113.5 (C), 52.9 (C), 41.9 (CH_2_), 20.5 (CH_3_), 12.7 (CH_3_), 11.3 (CH_3_).

*1-Propyl-2,3,3-trimethyl-5-sulfo-3H-indolium* (**16**): Iodopropane (0.20 mL, 2.1 mmol) and potassium 2,3,3-trimethyl-3*H*-indole-5-sulfonate (0.10 g, 0.36 mmol) in 1 mL of acetonitrile were added to a microwave reaction vial. The CEM microwave oven was set to 150 °C for 30 min with a ramp time of 1 min. After cooling, the product was concentrated under reduced pressure. The resulting red solid was stirred in acetone then filtered and washed with acetone and cold ether. The light pink solid was dried *in vacuo*, yielding 63% pure product. MP 208–211 °C; ^1^H-NMR δ (ppm): 8.03 (s, 1H), 7.95 (d, *J =* 8.38 Hz, 2H), 7.81 (d, *J =* 1.49 Hz, 2H), 4.44 (t, *J =* 7.69 Hz, 2H), 2.86 (s, 3H), 1.86 (m, 2H), 1.55 (s, 6H), 0.99 (t, *J =* 7.40 Hz, 3H); ^13^C-NMR δ (ppm): 195.7 (C), 149.3 (CH), 141.5 (CH), 140.9 (CH), 126.3 (C), 120.7 (C), 114.9 (C), 54.2 (C), 48.9 (CH_2_), 21.9 (CH_2_), 20.7 (CH_3_), 14.1 (CH_3_), 10.7 (CH_3_).

*1-Butyl-2,3,3-trimethyl-5-sulfo-3H-indolium* (**17**): Iodobutane (0.20 mL, 1.8 mmol) and potassium 2,3,3-trimethyl-3*H*-indole-5-sulfonate (0.10 g, 0.36 mmol) in 1 mL of acetonitrile were added to a microwave reaction vial. The CEM microwave oven was set to 155 °C for 35 min with a ramp time of 1 min. After cooling, the product was concentrated under reduced pressure then stirred in acetone until residue formed. The solution was decanted and the residue was suspended in acetone until solid product had further formed. The solid was filtered and washed with acetone and cold ether and dried *in vacuo*, yielding a red solid in 58% yield. MP 145–149 °C; ^1^H-NMR δ (ppm): 8.02 (s, 1H), 7.95 (d, *J =* 8.38 Hz, 1H), 7.81 (d, *J =* 1.34 Hz, 1H), 4.46 (t, *J =* 7.63 Hz, 2H), 2.87 (s, 3H), 1.82 (q, *J =* 7.08 Hz, 2H), 1.55 (s, 6H), 1.42 (q, *J =* 7.78 Hz, 2H), 0.93 (t, *J =* 7.36 Hz, 3H); ^13^C-NMR δ (ppm): 197.1 (C), 149.1 (CH), 141.4 (CH), 140.8 (CH), 126.2 (C), 120.5 (C), 114.9 (C), 54.1 (C), 47.5 (CH_2_), 30.6 (CH_2_), 29.0 (CH_2_), 21.8 (CH_2_), 19.2 (CH_3_), 14.0 (CH_3_), 13.4 (CH_3_).

*1-(2-Hydroxyethyl)-2,3,3-trimethyl-5-sulfo-3H-indolium* (**18**)*:* 2-bromoethanol (0.20 mL, 2.8 mmol) and potassium 2,3,3-trimethyl-3*H*-indole-5-sulfonate (0.10 g, 0.36 mmol) were added to a microwave reaction vial. The CEM microwave oven was set to 125 °C for 5 min with a ramp time of 1 min. After cooling, the product was stirred in 1:1:3 of chloroform/acetone/ether. The light grey solid was filtered and washed with cold ether and dried *in vacuo*, yielding 69% pure product. MP 255–260 °C; ^1^H-NMR δ (ppm): 8.03 (s, 1H), 7.94 (d, *J =* 8.38 Hz, 2H), 7.80 (d, *J =* 1.40 Hz, 2H), 4.61 (t, *J =* 4.84 Hz, 2H), 3.87 (t, *J =* 4.86 Hz, 2H), 2.85 (s, 3H), 1.56 (s, 6H); ^13^C-NMR δ (ppm): 198.1 (C), 149.1 (CH), 141.4 (CH), 141.0 (CH), 126.1 (C), 115.0 (C), 57.7 (C), 54.4 (C), 50.4 (CH_2_), 21.9 (CH_2_), 14.6 (CH_3_).

*1-(5-Carboxypentyl)-2,3,3-trimethyl-5-sulfo-3H-indolium* (**19**)*:* 6-Bromohexanioc acid (0.23 g, 1.2 mmol) and potassium 2,3,3-trimethyl-3*H*-indole-5-sulfonate (0.10 g, 0.36 mmol) in 1 mL of acetonitrile were added to a microwave reaction vial. The CEM microwave oven was set to 150 °C for 30 min with a ramp time of 1 min. After cooling for one hour, the vial was reintroduced to the microwave oven which was set to 150 °C for 15 min. After sitting and cooling for one hour, the precipitate was induced by agitation. The light pink solid was filtered and washed with 1:1 2-propanol/ether and dried *in vacuo*, yielding 72% pure product. MP 169–172 °C; ^1^H-NMR δ (ppm): 8.02 (s, 1H), 7.94 (d, *J =* 8.37 Hz, 1H), 7.81 (d, *J =* 1.31 Hz,1H), 4.46 (t, *J =* 7.41 Hz, 2H), 2.87 (s, 3H), 2.53 (q, *J =* 1.75 Hz, 2H), 2.22 (t, *J =* 7.20 Hz, 2H), 1.84 (q, *J =* 7.02 Hz, 2H), 1.55 (s, 6H), 1.41 (q, *J =* 7.03 Hz, 2H); ^13^C-NMR δ (ppm): 196.1 (C), 173.1 (C), 148.1 (CH), 140.3 (CH), 139.7 (CH), 125.1 (C), 119.5 (C), 113.7 (C), 53.0 (C), 46.3 (CH_2_), 32.1 (CH_2_), 25.6 (CH_2_), 24.1 (CH_2_), 22.8 (CH_2_), 20.6 (CH_3_), 12.9 (CH_3_).

*1-Butyl-2,3,3-trimethylindolium iodide* (**20**): 2,3,3-Trimethylindolenine (0.52 mL, 3.2 mmol) and iodobutane (0.76 mL, 6.6 mmol) were added to a microwave reaction vial. The microwave oven was set to 130 °C for 20 min with a ramp time of 2 min. After cooling, the product was stirred in acetone and ether. The yellow solid was filtered, washed with cold ether and dried *in vacuo,* yielding 70% pure product. MP 105-110 °C; ^1^H-NMR δ (ppm): 0.95 (t, *J* = 7.29 Hz, 3H), 1.44 (m, 2H), 1.55 (s, 6H), 1.83 (m, 2H), 2.87 (s, 3H), 4.47 (t, *J* = 7.72 Hz, 2H), 7.64 (m, 1H), 7.8 (m, 1H), 8 (m, 1H); ^13^C-NMR δ (ppm): 196.3 (C), 141.8 (CH), 141.0 (CH), 129.3 (C), 128.9 (C), 123.5 (C), 115.4 (CH), 54.1 (C), 47.4 (CH_2_), 29.2 (CH_2_), 22.0 (CH_2_), 19.3 (CH_3_), 14.1 (CH_3_), 13.6 (CH_3_).

## 4. Conclusions

The single mode microwave system has provided substantially decreased reaction times, simplicity of reaction procedure, and comparable or increased reaction yields observed for 20 heterocyclic salts. 
